# Development of KSHV vaccine platforms and chimeric MHV68-K-K8.1 glycoprotein for evaluating the *in vivo* immunogenicity and efficacy of KSHV vaccine candidates

**DOI:** 10.1128/mbio.02913-24

**Published:** 2024-10-30

**Authors:** Wan-Shan Yang, Dokyun Kim, Soowon Kang, Chih-Jen Lai, Inho Cha, Pei-Ching Chang, Jae U. Jung

**Affiliations:** 1 Department of Cancer Biology and Infection Biology, Lerner Research Institute, Cleveland Clinic, Cleveland, Ohio, USA; 2 Global Center for Pathogen Research and Human Health, Lerner Research Institute ,Cleveland Clinic, Cleveland, Ohio, USA; 3 Institute of Microbiology and Immunology, National Yang Ming Chiao Tung University, Hsinchu, Taiwan; 4 Institute of BioPharmaceutical Sciences, National Sun Yat-sen University, Kaohsiung, Taiwan; 5 Department of Molecular Biology and Microbiology, Case Western Reserve University School of Medicine, Cleveland, Ohio, USA; University of California, Davis, Davis, California, USA

**Keywords:** Kaposi's sarcoma-associated herpesvirus, MHV68, vaccines

## Abstract

Kaposi’s sarcoma-associated herpesvirus (KSHV)/human herpesvirus 8 is an etiological agent of Kaposi’s Sarcoma, multicentric Castleman’s disease, and primary effusion lymphoma. Considering the high seroprevalence reaching up to 80% in sub-Saharan Africa, an effective vaccine is crucial for preventing KSHV infection. However, vaccine development has been limited due to the lack of an effective animal model that supports KSHV infection. Murine Herpesvirus 68 (MHV68), a natural mouse pathogen persisting lifelong post-infection, presents a promising model for KSHV infection. In this study, we developed KSHV vaccine and a chimeric MHV68 carrying the KSHV glycoprotein, serving as a surrogate challenge virus for testing KSHV vaccines in a mouse model. Among KSHV virion glycoproteins, K8.1 is the most abundant envelope glycoprotein with the highest immunogenicity. We developed two K8.1 vaccines: K8.1 mRNA-lipid nanoparticle (LNP) vaccine and K8.1_26–87_-Ferritin (FT) nanoparticle vaccines. Both induced humoral responses in immunized mice, whereas K8.1 mRNA LNP also induced T cell responses. Using BACmid-mediated homologous recombination, the MHV68 M7 (gp150) gene was replaced with KSHV K8.1 gene to generate chimeric MHV68-K-K8.1. MHV68-K-K8.1 established acute and latent infection in the lungs and spleens of infected mice, respectively. Mice immunized with K8.1 mRNA LNP or K8.1_26–87_-FT showed a reduction of MHV68-K-K8.1 titer but not MHV68 wild type (WT) titer in the lung. In addition, viral reactivation of MHV68-K-K8.1 was also significantly reduced in K8.1 mRNA LNP-immunized mice. This study demonstrates the effectiveness of two vaccine candidates in providing immunity against KSHV K8.1 and introduces a surrogate MHV68 system for evaluating vaccine efficacy *in vivo*.

**IMPORTANCE:**

Kaposi’s sarcoma-associated herpesvirus (KSHV) is a prevalent virus that establishes lifelong persistent infection in humans and is linked to several malignancies. While antiretroviral therapy has reduced Kaposi’s Sarcoma (KS) complications in people with HIV, KS still affects individuals with well-controlled HIV, older men without HIV, and transplant recipients. Despite its significant impact on human health, however, research on KSHV vaccine has been limited, mainly due to the lack of interest and the absence of a suitable animal model. This study addresses these challenges by developing KSHV K8.1 vaccine with two platforms, mRNA lipid nanoparticle (LNP) and FT nanoparticle. Additionally, chimeric virus, MHV68-K-K8.1, was created to evaluate KSHV vaccine efficacy *in vivo*. Vaccination of K8.1 mRNA LNP or K8.1_26–87_-FT significantly reduced MHV68-K-K8.1 titers. Developing an effective KSHV vaccine requires an innovative approach to ensure safety and efficacy, especially for the immunocompromised population and people with limited healthcare resources. This study could be a potential blueprint for future KSHV vaccine development.

## INTRODUCTION

Kaposi’s sarcoma-associated herpesvirus (KSHV), also known as human herpesvirus 8, is one of the human oncogenic viruses. KSHV is associated with human malignancies such as Kaposi’s Sarcoma (KS), primary effusion lymphoma (PEL), and multicentric Castleman’s disease (1). The virus was first identified in a KS lesion of an AIDS patient in 1994 (2). While the KSHV-infected population is globally distributed, the occurrence of KS and KSHV-associated diseases is disproportionately enhanced under certain environmental factors, such as co-infection with other pathogens and compromised host immunity (3, 4). The prognosis of KSHV-related diseases, such as PEL, remains poor, with an overall survival of around 12 months despite clinical management or therapy (5). Given the high seroprevalence of up to 80% in specific geographic regions, an effective vaccine is a crucial and promising option to promote public health by preventing the spread of KSHV.

The life cycle of KSHV includes lytic and latent phases. Upon infection, KSHV typically maintains latency and expresses only a limited number of viral genes to sustain persistent infection and evade host immune detection (6). The current standard treatment for KSHV-related diseases is not entirely effective, partially due to viral evasion mechanisms (7, 8). Therefore, the objective of this study is to develop KSHV vaccines to reduce initial viral infection. Viral glycoproteins on the virion surface, gB, gH/gL, gM/gN, and K8.1, play crucial roles in viral entry and infection (9, 10). Among these, K8.1 has been shown in several studies to induce strong B and T cell responses (11–14) and plays a critical role in mediating B cell tropism during KSHV entry (15, 16). Due to its high immunogenicity, K8.1 was selected as the vaccine antigen in this study to target KSHV.

Various vaccine platforms exist for combating DNA viruses. In our study, we applied two different vaccine platforms to target KSHV K8.1, lipid nanoparticle (LNP)-encapsulated mRNA and self-assembling nanoparticle protein vaccines. During the COVID-19 pandemic, mRNA vaccines demonstrated efficacy through rapid development and adaptability to emerging variants (17). Previous studies have also shown vaccine efficacy of virally codon-optimized mRNA vaccine, providing enhanced immunity against wild type (WT) and Delta strains of SARS-CoV-2 (18). Similarly, self-assembling nanoparticle protein subunit vaccines have shown efficacy against viruses such as SARS-CoV-2, severe fever with thrombocytopenia syndrome virus, and Epstein-Bar virus, a gammaherpesvirus closely related to KSHV (18–22). Nanoparticle platforms, particularly those utilizing Ferritin (FT), leverage larger scaffold attachment for enhanced immune cell uptake and repetitive antigen arrays for efficient activation of multiple B cell receptors (23–25). Hence, both approaches, mRNA-LNP and FT nanoparticle, were employed in this study for K8.1-based vaccine development.

While previous research has identified potential KSHV vaccine candidates (26–29) inducing KSHV-specific immune responses, the lack of *in vivo* characterization of protective immunity against KSHV infection has limited the scope of these studies. To address this gap, our study utilized a chimeric murine gammaherpesvirus 68 (MHV68) bearing KSHV K8.1 (MHV68-K-K8.1) as a challenge virus in a mouse model to evaluate the protective efficacy of K8. vaccines. By replacing a functionally homologous gene on the MHV68 genome with KSHV K8.1, MHV68-K-K8.1 was able to establish acute infection and replication in mouse lung tissues and latent infection in mouse splenocytes. Immunization of mice with K8.1 mRNA-LNP and K8.1-FT vaccines induced robust immune responses, leading to reduced virus titers in lung tissue and impaired viral reactivation in splenocytes upon challenge with MHV68-K-K8.1. Overall, this study presents two vaccine candidates and an *in vivo* mouse model for assessing vaccine efficacy against KSHV.

## RESULTS

### Development of K8.1 vaccine candidates

**Fig 1:**
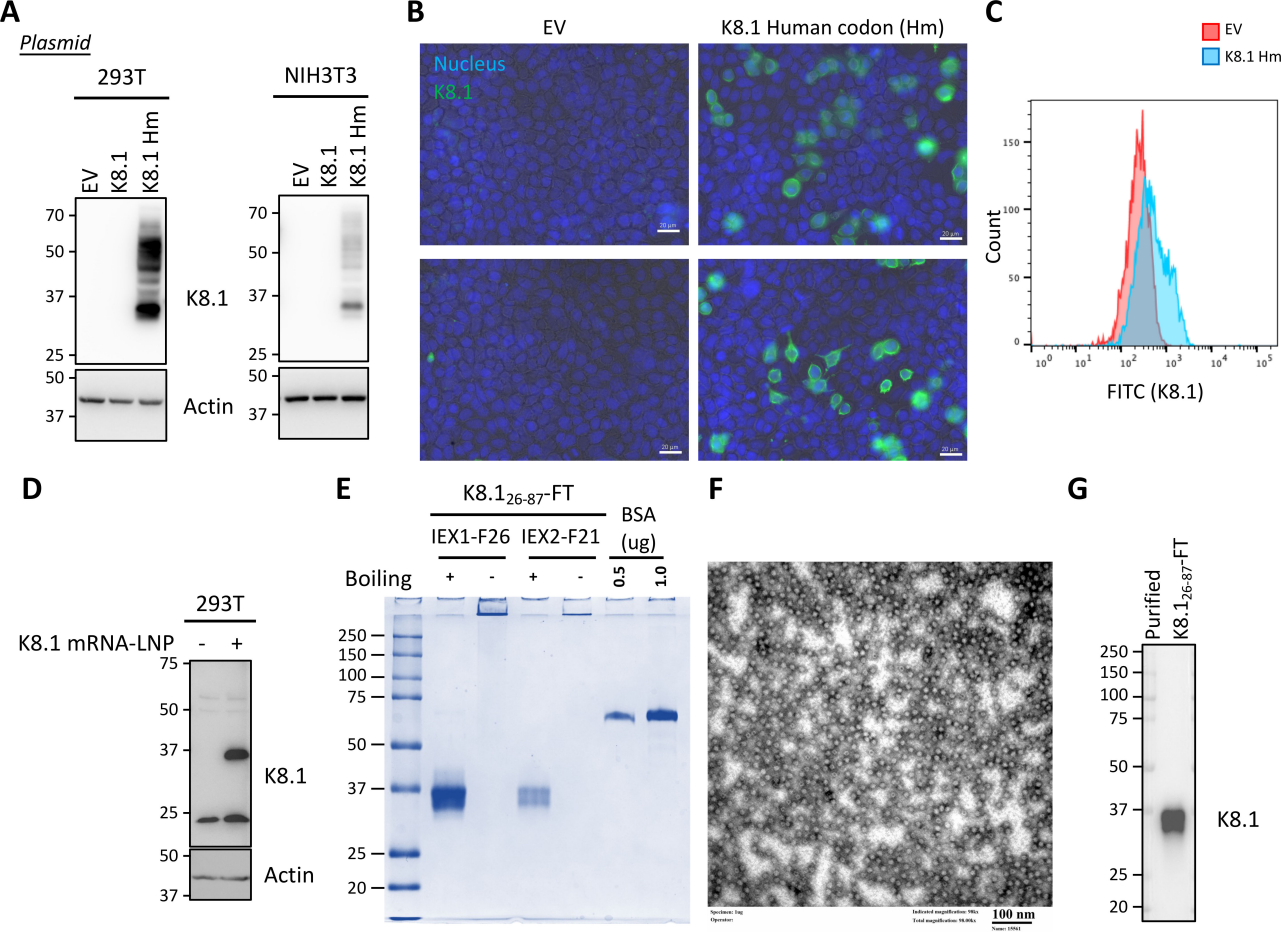
K8.1 vaccine generation. (**A**) HEK293T (left panel) or NIH3T3 (right panel) cells were transfected with plasmid for encoding K8.1 in original codon (K8.1) or human codon (K8.1 Hm). Cells transfected with empty vector (EV) was included as negative control. Transfected cells were lysed and analyzed by immunoblotting with anti-K8.1 and anti-Actin antibodies. (**B**) 293T cells were transfected with plasmid carrying K8.1 in human codon. Forty-eight hours after transfection, cells were fixed and stained with anti-K8.1 antibody as primary antibody and anti-mouse-FITC as secondary antibody. Slides were visualized using a fluorescent microscope. (**C**) HEK293T cells transfected with plasmid as described in (**B**) were analyzed by flow cytometry with anti-K8.1 antibody (#4A4) and FITC anti-mouse antibody. (**D**) HEK293T cells were transfected with LNP carrying K8.1 mRNA. Twenty-four hours later, the cells were lysed and analyzed as described in (**A**). (**E**) Immuno-dominant region of K8.1 fused with ferritin (K8.1_26–87_-FT) was purified from cultured medium of transfected HEK293T cell with ion exchange chromatography (IEX). Flowthrough from first round IEX (IEX1) were further purified with second round IEX (IEX2). Number 26 fraction (F26) in IEX1 and number 21 fraction (F21) in IEX2 were concentrated and buffer exchanged to phosphate buffered saline (PBS) with 100 kDa cutoff column. Final purified protein in sample buffer was analyzed for their molecular weights by SDS-PAGE without (“−” lane) or with (“+” lane) boiling at 95°C for 5 minutes. BSA of 0.5 and 1 µg was included as standards for protein quantification. (**F**) Purified K8.1_26–87_-FT at 250 ug/mL concentration was loaded to 300 mesh carbon grid and negatively stained by 1% uranyl acetate. Stained grid was observed by transmission electron microscopy. (**G**) Purified K8.1_26–87_-FT was analyzed by immunoblotting with anti-K8.1 antibody.

We developed mRNA vaccine and self-assembling nanoparticle vaccine carrying KSHV K8.1. Considering a previous study in which K8.1 in the original viral codon showed low translation efficiency (30), we synthesized a human codon-optimized K8.1 sequence for cloning into the mRNA expression construct. We compared the expression level of K8.1 in the original viral codon and human codon in transfected HEK293T or NIH3T3 cells. Human codon-optimized K8.1 showed a higher expression level than the original viral codon (Fig. 1A). We also checked K8.1 surface expression by fluorescence microscopy (Fig. 1B) and flow cytometry (Fig. 1C) to demonstrate K8.1 surface presentation, enabling recognition by immune cells after vaccine delivery. Additionally, we generated K8.1 mRNA with human codon via *in vitro* transcription. To deliver the mRNA into cells, it was encapsulated in LNP, and the delivery was confirmed via immunoblotting of HEK293T cell lysates transfected with mRNA-LNP, demonstrating efficient expression of K8.1 (Fig. 1D).

In addition to the mRNA- LNP vaccine, we generated an FT-based K8.1 nanoparticle vaccine. Our previous study demonstrated that FT-fused antigen nanoparticle vaccines induce stronger immunogenicity than soluble protein alone (19, 22, 31, 32). Therefore, we utilized the immunodominant region (amino acid residues from 26 to 87) of K8.1 (14, 33) fused with FT (K8.1_26–87_-FT) in a mammalian expression vector for protein vaccine purification from transiently transfected HEK293T cells. K8.1_26–87_-FT nanoparticle was purified via ion exchange chromatography from the transfected cell culture medium and evaluated for its purity by SDS-PAGE with or without boiling at 95°C. With boiling, the purified sample showed the expected molecular weight of K8.1_26–87_-FT monomer, including the glycosylation on K8.1 (Fig. 1E). Without boiling, the purified protein maintained the higher order particle structure that composed by FT 24 subunits, as demonstrated by minimal migration in SDS-PAGE (Fig. 1E). This implies that K8.1_26–87_-FT vaccines are stable under room temperature which remained as complex architecture even in the presence of detergent. The structure would be denatured with heating condition. In the next step, to visualize the nanoparticle structure of purified K8.1_26–87_-FT, we performed negative staining and checked with transmission electron microscopy (TEM), showing spherical nanoparticles (Fig. 1F), as seen in our previous FT-based nanoparticles (22). Lastly, the presence and antigenicity of K8.1 from the purified K8.1_26–87_-FT nanoparticle were analyzed by immunoblotting with anti-K8.1 antibody (Fig. 1G).

### K8.1 vaccine immunization and host immune responses

**Fig 2:**
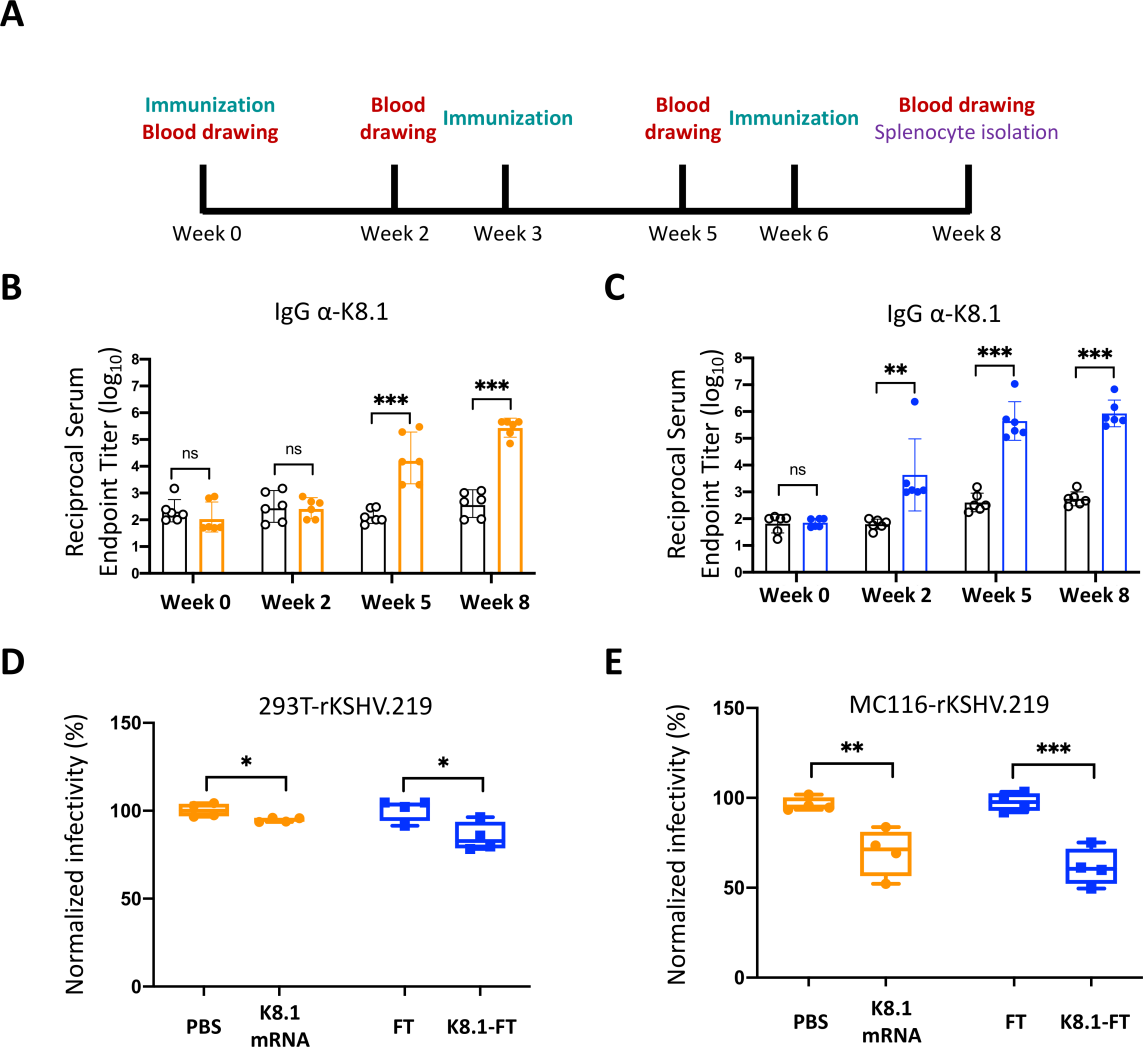
K8.1 vaccine immunization and B cell response *in vivo*. (**A**) Six Balb/c female mice were immunized at weeks 0, 3, and 6. Blood collection for serum isolation was performed at weeks 0, 2, 5, and 8. (**B**) Anti-K8.1 antibody titers in mouse serum were measured by ELISA. Titers in PBS are shown as empty dots, and those in K8.1 mRNA immunized group are shown as colored solid dots. (**C**) Anti-K8.1 antibody titers in mouse serum were measured by ELISA. Titers in FT group are shown as empty dots, and those in K8.1_26–87_-FT immunized group are shown as colored solid dots. (**D and E**) Neutralization activity of serum from mouse described in (**A**) was determined by rKSHV.219 infectivity in HEK293T cells (**D**) or MC116 cells (**E**). Infectivity was determined by flow cytometry and normalized using control group as 100% infection. Data points from four mice were included in each group. Data from six mice were presented in (**B and C**). Data from four mice were presented in (**D and E**).

To investigate whether K8.1 mRNA and K8.1_26–87_-FT vaccines induce immune responses, we immunized Balb/c mice with K8.1 vaccine at weeks 0, 3, and 6. Anti-K8.1 antibody titers in mouse sera were quantified by enzyme-linked immunosorbent assay (ELISA) at weeks 0, 2, 5, and 8 (Fig. 2A). Anti-K8.1 antibody titers increased immediately at weeks 2, 5, and 8 in K8.1_26–87_-FT immunized mice (Fig. 2C), whereas they robustly increased only at weeks 5 and 8 in K8.1 mRNA-immunized mice (Fig. 2B). To further examine whether anti-K8.1 antibodies in mouse serum show neutralization activity to block virus infection, we performed neutralization assays using rKSHV.219 for the infection of HEK293T epithelial cells or MC116 B cells. Because rKSHV.219-infected cells express green fluorescent protein (GFP) (34), we analyzed the neutralization activity by flow cytometry. Diluted sera from K8.1 mRNA-immunized mice or K8.1_26–87_-FT-immunized mice reduced KSHV infectivity in MC116 B cells by approximately 30% or 40%, respectively, although they showed weak neutralizing activity in HEK293T epithelial cells (Fig. 2D and E). Neutralization activity from both vaccines was stronger in MC116 cells than in HEK293T cells, which may be attributable to a difference in the virological significance of K8.1 in KSHV infection (15, 16). In addition, neutralization activity was slightly higher in K8.1_26–87_-FT-immunized mice sera, implying that the FT-based vaccine induces a more potent antibody response than mRNA vaccine.

**Fig 3:**
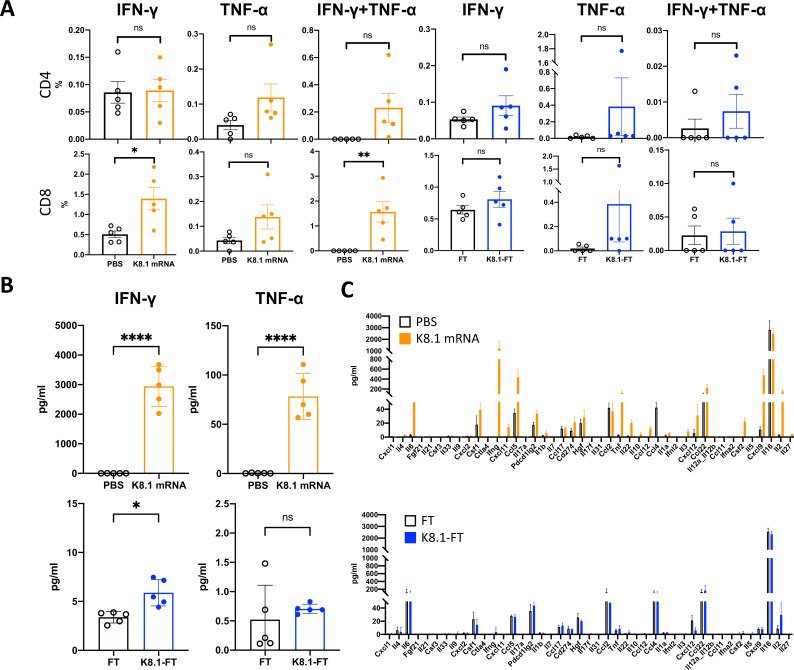
K8.1 vaccine immunization and T cell response *in vivo*. (**A**) Splenocyte from immunized mouse as described in Fig. 2A was isolated at week 8. Splenocyte was *ex vivo* stimulated with K8.1 OLP pool in the presence of Brefeldin A for 12 hours. Surface markers of CD3, CD4, and CD8 were stained using respective Fluorescence-activated Cell Sorting (FACS) antibodies before permeabilization and intracellular cytokines staining for IFN-γ and TNF-α. Stained splenocyte were analyzed by flow cytometry. (**B**) Splenocytes harvested from mouse as described in (**A**) were *ex vivo* stimulated with K8.1 peptide pool for 12 hours. IFN-γ and TNF-α secretion in the culture medium was analyzed by ELISA. (**C**) Culture medium from splenocytes described in (**B**) was harvested and subjected to multiplex cytokine array analysis. Data from five mice were presented in (A–C).

In addition to the vaccine-induced humoral immunity, we also investigated T cell responses from splenocytes harvested at week 8 (Fig. 2A). After 12 hours of *ex vivo* splenocyte stimulation with the K8.1 overlapping peptide (OLP) pool, we stained splenocytes with antibodies of T cell surface markers, including CD3, CD4, and CD8 to distinguish subtypes of T cells. We also performed intracellular antibody staining to investigate the expression levels of IFN-γ and TNF-α, which play critical roles in the anti-viral response (35–37). T cell population was first identified by CD3^+^ population which was further divided into CD4^+^ helper T cells and CD8^+^ cytotoxic T cells. Among two different type of T cells, we also characterized their intracellular cytokine, IFN-γ and TNF-α, expression levels. Among the CD3^+^ T cells, IFN-γ^+^ or IFN-γ^+^/TNF-α^+^ populations were significantly increased in CD8^+^ T cells but showed slight enhancement in TNF-α^+^ or IFN-γ^+^/TNF-α^+^ in CD4^+^ T cells, without significant difference in K8.1 mRNA-immunized group (Fig. 3A, left panel). Surprisingly, cytokine production did not significantly increase in the K8.1_26–87_-FT-immunized group (Fig. 3A, right panel).

In addition, secreted IFN-γ and TNF-α in culture medium after stimulation were also analyzed by ELISA, and results also demonstrated that higher cytokine secretion level was detected in K8.1 mRNA immunized group compared to K8.1-FT immunized group (Fig. 3B). Interestingly, secreted IFN-γ in the culture medium significantly increased in the K8.1_26–87_-FT-immunized group (Fig. 3B, bottom panel). This suggests that although IFN-γ production by T cells did not increase in the K8.1_26–87_-FT-immunized group, there might be other cell types in the spleen secreting IFN-γ after K8.1 OLP stimulation (36). Finally, the supernatants of OLP-stimulated splenocytes from immunized mice were subjected to a multiplex cytokine array. These results also showed that K8.1 mRNA LNP immunization, but not K8.1_26–87_-FT-immunization, strongly induced the productions of many cytokines and chemokines upon K8.1 OLP stimulation, showing robust immune responses were induced K8.1 mRNA-immunized group (Fig. 3C). These results suggest that weaker cellular responses in the K8.1_26–87_-FT-immunized group which might be attributable to the differences between the antigens covered by K8.1_26–87_-FT and the full-length K8.1 peptide pool used for the *ex vivo* stimulation. This finding also corresponds to a previous study demonstrating that protein vaccines tend to induce a low T cell response (38).

### Generation of MHV68-K-K8.1

**Fig 4:**
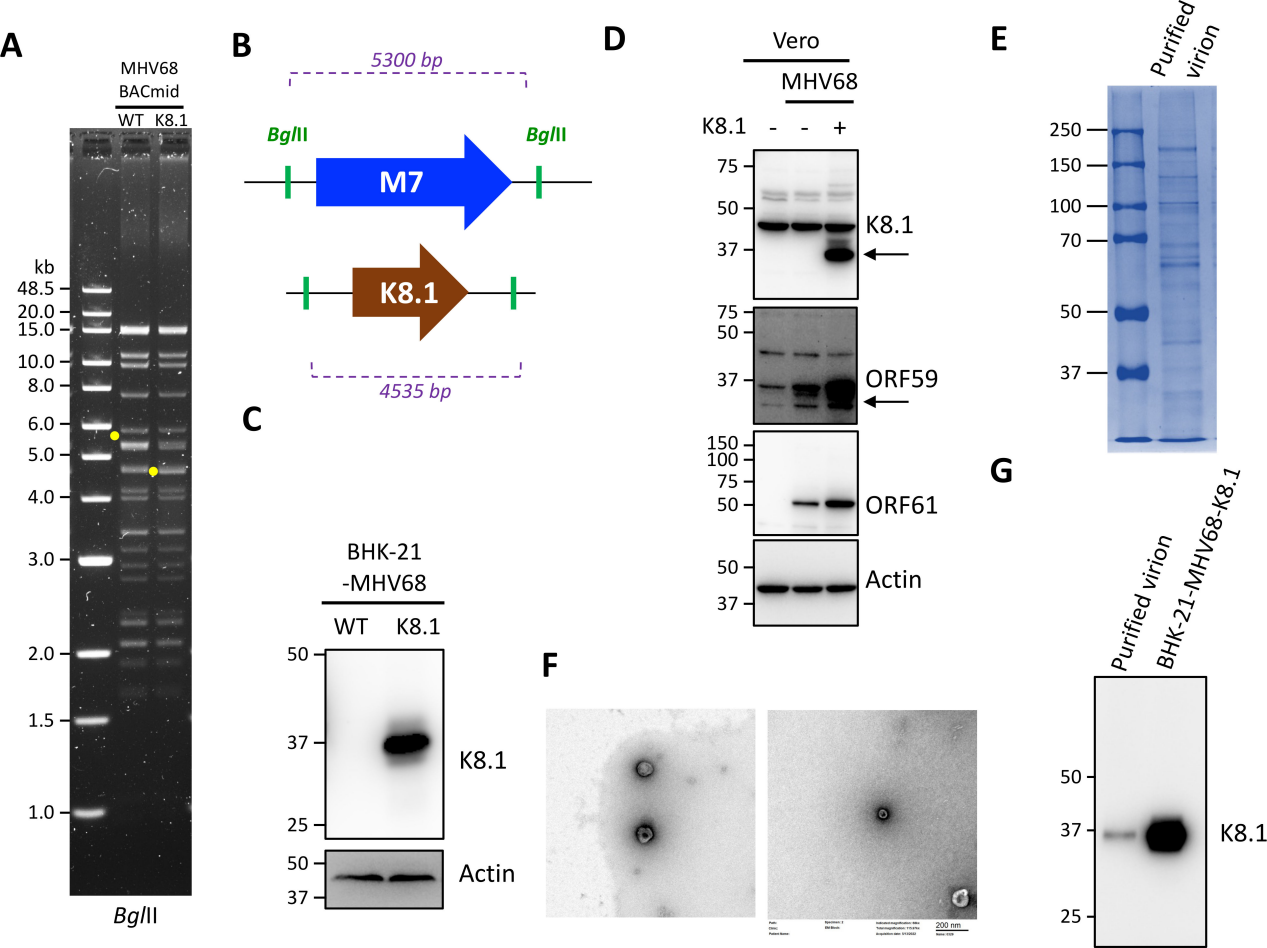
Recombinant MHV68-K-K8.1 generation. (**A and B**) MHV68 WT BACmid was engineered by replacing M7 with KSHV K8.1 by Red-mediated recombination in *Escherichia coli* GS1783. MHV68 WT BACmid or -K8.1 BACmid was extracted and digested with *Bgl*II. Digested BACmid was separated by 0.7% agarose gel electrophoresis. Fragment with M7 in MHV68-WT BACmid and fragment with K8.1 in MHV68-K-K8.1 BACmid are indicated in yellow dots on the gel (**A**). The fragments sizes are 5,300 and 4,535 bp, respectively, as shown in (**B**). (**C**) BHK-21 cells were transfected with BACmid described in (**A**). Cell lysates from transfected BHK-21 were analyzed by immunoblotting with anti-K8.1 (#4A4) and anti-Actin antibodies. (**D**) Vero E6 cells infected with virus described in (**C**) were analyzed by immunoblotting with anti-K8.1, MHV68 ORF59, and MHV68 ORF61 antibodies. Actin expression was also analyzed as loading control. (**E**) Virus from (**D**) was purified using 15%–50% continuous sucrose gradient ultracentrifugation. Purified virions were separated by SDS-PAGE and subjected to Coomassie blue staining. (**F**) Purified virions in (**E**) were fixed, stained, and observed under a transmission electron microscope. (**G**) Purified virion in (**E**) was mixed with sample buffer and analyzed by immunoblotting using anti-K8.1 antibody.

To test vaccine-induced immunity *in vivo*, we engineered a chimeric murine gammaherpesvirus, MHV68, by replacing M7 with KSHV K8.1 via Red-mediated homologous recombination, where MHV68 M7 is homologous to KSHV K8.1. We verified the resultant sequence by Sanger sequencing and confirmed the genome structure of the recombinant BACmid by restriction enzyme digestion. After *Bgl*II digestion, the 5.3 kb band in MHV68-WT BACmid migrated to 4.5 kb in MHV68-K8.1 BACmid (Fig. 4A and B).

**Fig 5:**
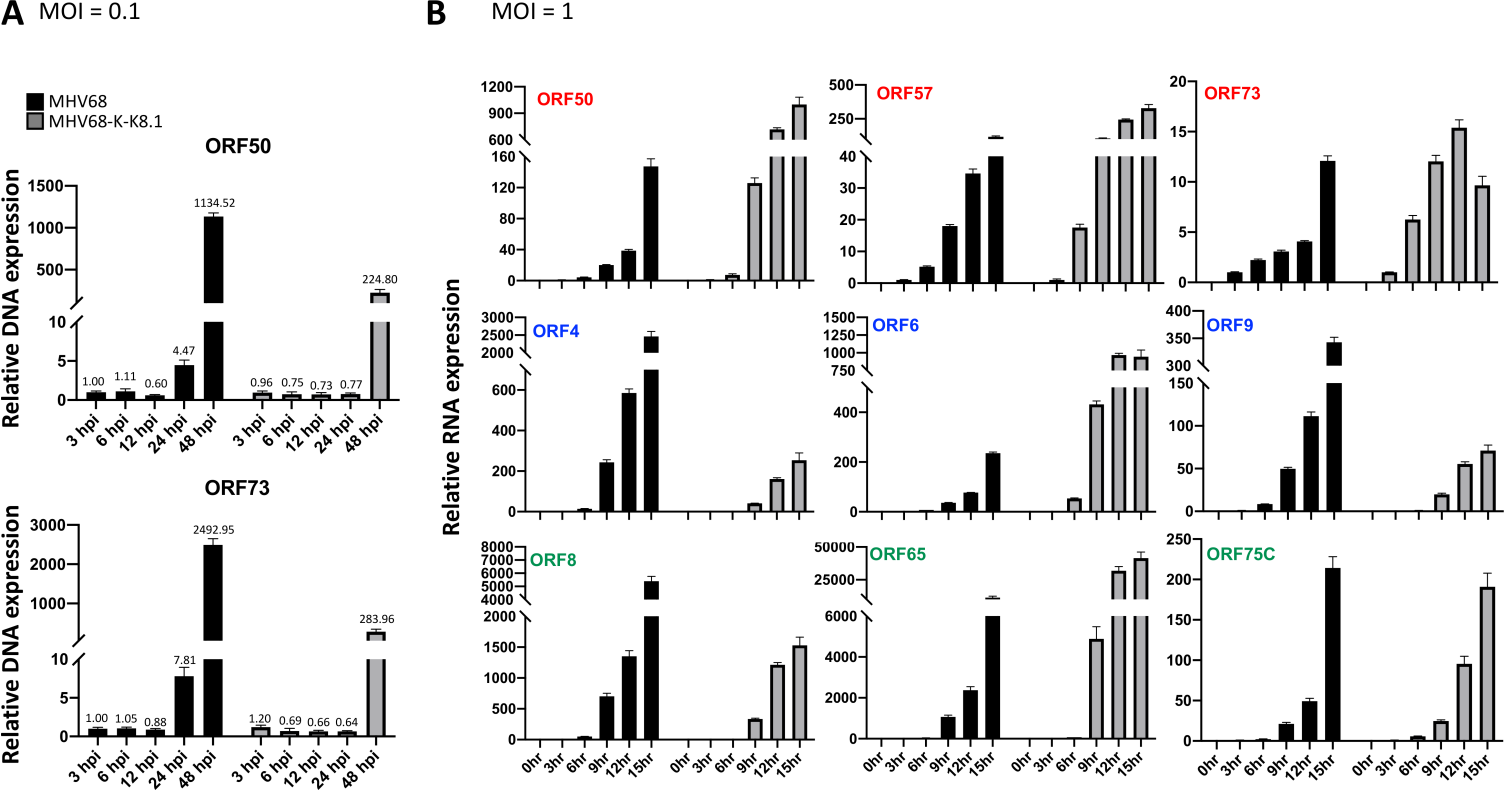
Recombinant MHV68-K-K8.1 viral gene transcription. (**A**) NIH3T3 cells were infected with multiplicity of infection (MOI) = 0.1 of MHV68-WT or -K-K8.1 and harvested at 3, 6, 12, 24, and 48 hours post infection (hpi) for genomic DNA extraction. DNA copies were quantified with real-time quantitative PCR (qPCR) with primer pairs targeting MHV68 ORF50 and ORF73 (sequences provided in Table S2). Quantification result of mouse L8 gene was included as internal control for relative expression level determination. DNA copies were presented as relative copies to MHV68 WT 3 hpi and showed on top of bar charts. (**B**) NIH3T3 cells were infected with MOI = 1 of MHV68-WT or -K-K8.1 and harvested at 3, 6, 9, 12, and 15 hpi for RNA extraction and reverse transcription. Viral gene expression level was determined by real-time qPCR with primer pairs targeting MHV68 genes as indicated. Quantification result of mouse L8 gene was included as internal control for relative expression level determination. Genes labeled with red, blue, and green represent for immediate early, early, and late genes. Relative RNA expression was presented as comparing to the detectable expression in the earliest time point of viral genes which sets as 1. Each point is the average of three technical replicates.

To examine whether the engineered virus expressed KSHV K8.1, we utilized BHK-21 cells for BACmid transfection to produce the chimeric virus. Cell lysates showed K8.1 expression only upon transfection with the recombinant BACmid (Fig. 4C). We further amplified the chimeric virus in Vero E6 cells and observed that MHV68 ORF59 and ORF61 expressions were not affected by the BACmid recombination (Fig. 4D). In addition, we purified virions by sucrose gradient ultracentrifugation (Fig. 4E) and demonstrated the intact virion structure under TEM (Fig. 4F). Immunoblotting of purified virions also showed that KSHV K8.1 was incorporated into the recombinant virion particles (Fig. 4G). We further checked viral replication of chimeric virus in cell culture system. Comparing to MHV68 WT, MHV68-K-K8.1 showed lower replication efficiency in NIH3T3 cells (Fig. 5A).

We further assessed the viral replication of the chimeric virus in a cell culture system. Compared to MHV68 WT, MHV68-K-K8.1 showed lower replication efficiency in NIH3T3 cells (Fig. 5A). To determine whether this reduced replication was due to differences of viral genes transcription after replacing MHV68 M7 with KSHV K8.1, we examined expression levels of viral immediate early (ORF50, ORF57, and ORF71), early (ORF4, ORF6, and ORF9), and late (ORF8, ORF65, and ORF75C) genes after 3, 6, 9, 12, and 15 hours infection. The results showed that the expression levels of MHV68-K-K8.1 genes were similar to those of MHV68 WT, with minor variations (Fig. 5B). Therefore, the lower level of MHV68-K-K8.1 replication (Fig. 5A) does not appear to be due to reduced viral genes expressions but may be linked to deficiencies in virion assembly in MHV68-K-K8.1.

### K8.1 vaccines provide immunity against MHV68-K-K8.1 infection *in vivo*

**Fig 6:**
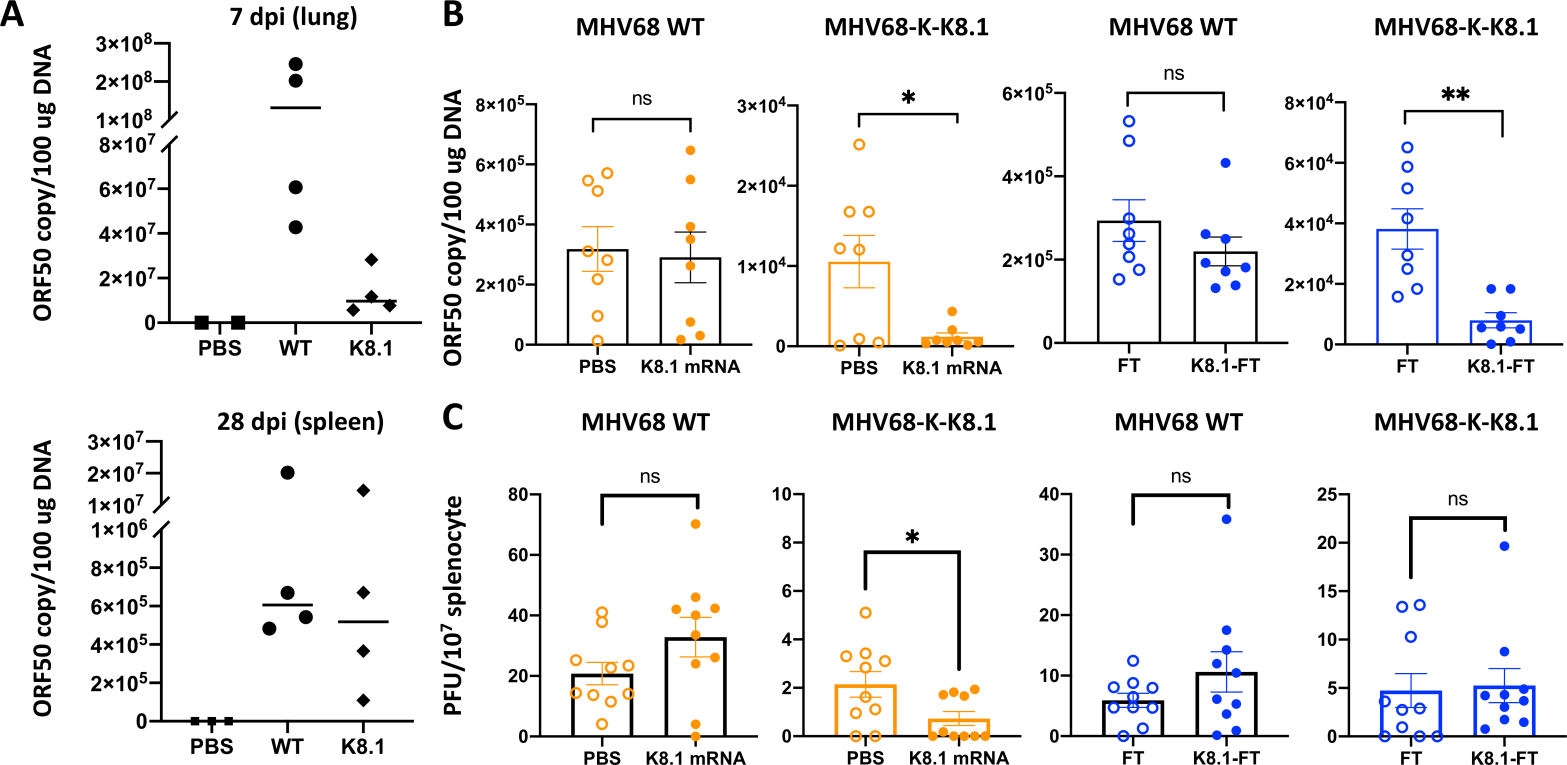
Protection effect of K8.1 immunization from MHV68-K-K8.1 challenge *in vivo*. (**A**) Four Balb/c mice were challenged with 500 plaque-forming unit (PFU)/mice of MHV68-WT or -K-K8.1 for 7 and 28 days and determined virus titer in lung and splenocyte by real-time qPCR with MHV68 ORF50 targeting primer. Quantification result of serially diluted pUC57-MHV68 ORF50 plasmid served as standard. (**B**) Total 10 immunized mice as described in Fig. 2A were challenged with MHV68 WT or MHV68-K-K8.1 at week 9 with 10^4^ PFU/mice. One week post infection, lung tissue was harvested, and MHV68 WT and MHV68-K-K8.1 titers were determined by real-time qPCR as described in (**A**). Results from 8 mice were presented. (**C**) Total 10 immunized mice as described in Fig. 2A were challenged with MHV68 WT or MHV68-K-K8.1 at week 9 with 10^4^ PFU/mice. Splenocyte were harvested and serially diluted for co-culturing with Vero E6 cells at 4 weeks post infection. Reactivated virus titer was quantified by PFU. Results from 10 mice were presented.

To make sure MHV68-K-K8.1 is applicable to the *in vivo* system for challenging study, we intranasally inoculated MHV68-K-K8.1 in mouse and found out that viral titer was detectable in lung at 7 days post infection (dpi) (Fig. 6A, top panel). In addition, MHV68-K-K8.1 also successfully established latent infection in splenocytes at 28 dpi (Fig. 6A, bottom panel). These indicate that while MHV68-K-K8.1 has lower efficiency in viral replication, it still retains the capability to establish latent viral infection in a mouse model.

Based on the efficient induction of humoral and cellular immunity following immunization with K8.1 vaccine candidates (Fig. 2 and 3), we investigated if they provide strong immunity against MHV68-K-K8.1 infection. MHV68 WT, which lacks KSHV K8.1, was included as a negative control, lacking KSHV K8.1. Two weeks after the last immunization, mice were intranasally challenged with MHV68 WT or MHV68-K-K8.1 in serum-free Dulbecco’s modified Eagle medium [DMEM; 1 × 10^4^ plaque-forming unit (PFU)/mouse]. After 4–7 dpi, mouse lungs were harvested to determined viral titers using real-time quantitative PCR of genomic DNAs. K8.1 mRNA or K8.1_26–87_-FT immunization considerably lowered MHV68-K-K8.1 DNA copy numbers (Fig. 6B). In addition, ORF61 immunohistochemistry (IHC) of the lung tissues from K8.1 vaccines immunized mice showed markedly reduced expression in MHV68-K-K8.1-infected mice (SI Appendix, Fig. S1). To further test whether K8.1 vaccinations affects viral latency, we examined the reactivation frequency of MHV68 WT or MHV68-K-K8.1 in mouse splenocytes 1 month after virus infection. The results showed that the K8.1 mRNA-immunized group had a lower reactivation frequency of MHV68-K-K8.1 but not MHV68 WT (Fig. 6C). Interestingly, K8.1_26–87_-FT immunization showed no effect on the reactivation frequency of either MHV68 WT or -K-K8.1 (Fig. 6C), which may correlate with the weaker T cell response elicited by K8.1_26–87_-FT immunization (Fig. 3A). This study demonstrates that K8.1 mRNA or K8.1_26–87_-FT vaccination markedly reduces MHV68-K-K8.1 titers in lung tissues, and only K8.1 mRNA vaccination can reduce the reactivation frequency of MHV68-K-K8.1 in splenocytes.

## DISCUSSION

KSHV is a human oncogenic virus associated with KS and B cell lymphoma. Current treatments for KSHV-associated diseases include surgery, chemotherapy, immunotherapy, and anti-viral drug (8, 39); however, these treatments have failed to clear KSHV. This failure might be attributed to KSHV’s manipulation of host gene expression (40) and mutations in the viral genome during long-term treatments (41, 42), which result in drug resistance. Given the limitations of current therapies, an effective method to block initial infection with KSHV is one of the most promising strategies to improve public health against KSHV.

In this study, we designed a KSHV vaccine targeting the most immunogenic glycoprotein, K8.1, using two different vaccine platforms: K8.1 mRNA-encapsulated LNP and K8.1_26–87_-FT self-assembling nanoparticles. K8.1 is a glycoprotein expressed on the viral surface during the lytic phase of KSHV infection. Previous studies show that K8.1 plays a significant role in viral entry by associating with heparan sulfate (43, 44) and facilitating other glycoproteins, such as gB and gH/gL, in recognizing their receptors on the host cell surface for viral entry (9, 10). Although KSHV utilizes multiple glycoproteins for the entry process, recent studies report that K8.1 contributes to KSHV infection in a cell-type-specific manner (15, 16). Since K8.1 is one of the most immunogenic antigens among viral glycoproteins (13, 14), both mRNA and FT nanoparticle vaccines induced a strong antibody response *in vivo* (Fig. 2B through E). The K8.1 mRNA vaccine successfully induced T cell responses in immunized mice, whereas the K8.1_26–87_-FT vaccine induced only minimal T cell responses (Fig. 3A). This may be associated with different K8.1 regions included in the two vaccine platforms; the mRNA vaccine covers the full length (228 amino acids) of K8.1, while the FT nanoparticle vaccine covers only the immunodominant region of K8.1 (14). Since full-length K8.1-FT was unstable, the segment of K8.1 comprising 26–87 aa, which covers a major immunodominant region, was fused with FT. This suggests that the minimal T cell responses observed with the K8.1_26–87_-FT vaccine were due to only parts of the K8.1 overlapping peptide pool successfully stimulating splenocytes *ex vivo* from K8.1_26–87_-FT-immunized mice. In addition, it has been indicated that mRNA vaccines can expand T cell population which is critical for clearing primary infection and suppressing persistent infection (38, 45, 46), implying that mRNA vaccine may provide long-term protection effect, as shown in Fig. 6C. In contrast, protein-based vaccine generally induces weaker CD8^+^ T cell response (47). Therefore, further studies are needed to improve cellular immune responses induced by protein-based vaccine, potentially through the use of different adjuvants.

Several studies have reported the immunogenicity of KSHV vaccines immunogenicity in *in vivo* mouse model (12, 26–29), where the neutralization activity of those vaccines was primarily measured by *in vitro* cell culture systems. In addition, the study of vaccine-mediated protection efficacy has been predominantly performed in humanized mouse model (21, 48, 49). While humanized mice offer significant advancements for immune response research, they still have certain limitations. Even the most advanced humanized mice do not fully replicate a normal immune system; their immune systems often remain immature or functionally compromised compared to a fully developed human immune system; the level of human-derived cell reconstitution is low; and the lifespan of human-derived T cells is short. To address this limitation, we developed a chimeric MHV68 carrying the replacement of KSHV glycoprotein as a surrogate challenge virus to evaluate the immunogenicity and efficacy of KSHV vaccine candidates in an *in vivo* WT mouse model. Using the MHV68 BAC, we created chimeric MHV68-K-K8.1, which carries the replacement of its M7 with the K8.1. Although the MHV68-K-K8.1 replicates at a slower rate, it retains the capability for lytic replication and establishes viral latency in infected mice. K8.1 mRNA or K8.1_26–87_-FT immunization considerably lowered the DNA copy numbers and viral gene expressions of MHV68-K-K8.1, but not MHV68 WT, in lung tissues (Fig. 6B). On the other hand, reactivation virus titers were significantly lower in K8.1 mRNA-immunized group, but not in K8.1_26–87_-FT-immunized group (Fig. 6C). This differences in reactivation frequency may be due to the stronger cellular immunity induced in the K8.1 mRNA vaccination. Overall, we demonstrate that MHV68-K-K8.1 serves as a surrogate virus to evaluate the effectiveness of KSHV vaccine candidates in a mouse model.

The goal of this study is twofold: first, to test two different K8.1 vaccine platforms against KSHV infection, and second, to develop chimeric MHV68-K-K8.1 as a surrogate challenge virus for testing the efficacy of KSHV vaccines in an *in vivo* mouse model. Although both K8.1 mRNA LNP and K8.1-FT vaccinations significantly hindered MHV-68-K-K8.1 infection in the lungs and splenocytes, they only partially blocked MHV68-K-K8.1 infection *in vivo*. This suggests that vaccination with K8.1 alone is not sufficient to provide robust immunity against KSHV infection, and other viral glycoproteins, such as gB and gH/gL, need to be included in future vaccine designs. Besides a strong B cell-mediated humoral response, a T cell-mediated cellular response also plays critical roles in the clearance of latently infected MHV68 (50).

One of the main difficulties in developing a herpesvirus vaccine is the ability of herpesviruses to establish latency. Latency is a state where the virus remains dormant in the host cells, often for the host’s lifetime, without producing new viral particles. This latency poses several challenges such as viral reservoirs, immune evasion, reactivation, and long-term immunity. An ideal vaccine would need to both prevent initial infection and control latent reservoirs to prevent reactivation. These challenges make herpesvirus vaccine development particularly complex compared to other viruses that do not establish latency.

In summary, this study highlights the characteristics of our two vaccine platforms and the suitability of our chimeric MHV68-K-glycoprotein challenge virus models for investigating the immunogenicity and efficacy of KSHV single and multivalent vaccine candidates in an *in vivo* mouse model.

## MATERIALS AND METHODS

### Mice experiment

Five-week-old female BALB/c mice were purchased from Jackson laboratory. All mouse experiments were performed in accordance with Institutional Animal Care and Use Committee approved protocol (Protocol number 2427). Animals were quarantined for 1 week before starting the experiment and housed at Cleveland Clinic Lerner Research Institute’s Biological Resource Unit equipped with 12 hours dark/light cycle throughout the experiment.

### Cell line

HEK293T, iSLK.219, NIH3T3, BHK-21, and Vero E6 were cultured in DMEM (Gibco) containing 10% fetal bovine serum (Gibco, FBS) and 1% penicillin/streptomycin (Gibco) at 37°C and 5% CO_2_. MC116 cells were cultured in Roswell Park Memorial Institute 1640 (Gibco) supplied with 10% FBS and 1% penicillin/streptomycin at 37°C. For KSHV selection, puromycin (Gibco) was added into culture medium of iSLK.219 to the concentration of 10 ug/mL. For rKSHV.219 production, iSLK.219 was treated with doxycycline at the concentration of 1 µg/mL for 5 days. KSHV was harvested following a previous publication (28). MHV68 was amplified via infection of Vero E6 cells, and the supernatant was centrifuged to remove cell debris and harvest the virus. Virus was stored at −80°C until further use.

### BACmid engineering

BACmid engineering was performed as described in a previous manuscript (51). Detailed procedure is described in SI Appendix, Supplementary methods.

### Plasmid

K8.1 original codon was PCR-amplified from pcDNA3-myc-K8.1 and subcloned to pZMV (18) vector (pZMV-K8.1). Full-length K8.1 in human codon (K8.1 Hm) was synthesized (GeneScript) and cloned into pZMV (pZMV-K8.1 Hm) in the identical method. For protein subunit vaccine, K8.1_26–87_-FT expression construct was PCR-amplified from pZMV-K8.1 Hm and subcloned into modified pFUSE (Invivogen) vector as described in our previous publication (22, 52). K8.1_26–198_-Fc expression construct was also PCR-amplified from pZMV-K8.1 Hm and subcloned into a commercially available pFUSE vector.

### Transmission electron microscopy

Two microgram of purified proteins was loaded to glow-discharged formvar film (Electron Microscopy Science) and incubated for 1 minute. Sample-adsorbed grids were washed by filtered 5 mM Tris-HCl (pH 7.0) and distilled water. Residual water was removed by blotting against filter paper, and the grid was stained with 1% uranyl formate (Electron Microscopy Science) for 1 minute. Images were collected with FEI Tecnai G2 Spirit BioTwin TEM.

### Virion purification with sucrose gradient ultracentrifugation

A detailed procedure is described in SI Appendix, Supplementary methods. In short, Vero E6 cells were infected with MHV68 WT or MHV68-K-K8 for 2–4 days and until 90% cytopathic effect was observed. Cell debris was removed, and harvested viruses were loaded onto sucrose gradient mixture for separation by centrifugation at 24,000 rpm.

### K8.1 mRNA vaccine generation

K8.1 mRNA generation followed our previous study (18) and described in detail SI Appendix, Supplementary methods. In brief, K8.1 mRNA was generated from linearized pZMV-K8.1 (or pZMV-K8.1 Hm) by using T7 RNA synthesis kit (New England Biolabs). DNA template was digested after transcription by DNase I, and RNA was purified by Monarch RNA cleanup kit (New England Biolabs). Linear mRNA was enzymatically polyadenylated with *E. coli* Poly (A) polymerase kit (New England Biolab) and encapsulated in LNP.

### K8.1_26–87_-FT vaccine and K8.1_26–198_-Fc purification

Detailed procedure was described in SI Appendix, Supplementary methods. Briefly, HEK293T cells were transfected with protein expression construct carrying signal peptide for secretion into the culture media. After harvesting medium, cell debris was removed by centrifugation before further purification by Akta pure chromatography system (Cytiva) or protein A/G Agarose beads (Thermo Fisher).

### Mouse immunization

Six- to 8-week-old mouse was intramuscularly immunized with 1 µg of mRNA vaccine diluted in PBS or 1 µg protein vaccine mixed with equal volume of AddaVax adjuvant (InvivoGen). Mouse blood was collected through retro-orbital route and incubated at room temperature for 1–3 hours to remove blood clotting. Isolated serum was heated at 55°C for 30 minutes and kept in −20°C until further characterization. Eight weeks after the last immunization, splenocyte were isolated and frozen in FBS with 10% (vol/vol) dimethyl sulfoxide (MP biomedical).

### Mouse viral challenging with MHV68-K-K8.1

Mice was anesthetized by ketamine/xylazine (100 mg/kg; 10 mg/kg) mixture in PBS (Gibco) and intranasally inoculated with MHV68 WT or MHV68-K-K8.1 in serum-free DMEM (1 × 10^4^ PFU/mice). One week after infection, a group of mice were sacrificed to harvest lung tissues, and the tissues were separated into two fractions for freezing at −80°C and fixation with 4% paraformaldehyde. Fixed tissues were embedded in paraffin and sectioned. Remaining mice were sacrificed 3–4 weeks after viral challenge, and splenocyte was isolated and made serial dilution for overnight co-culturing with Vero E6 cells to perform reactivation assay.

### Flow cytometry

Cells were harvested and washed in FACS buffer (PBS supplied with 2% FBS, 1 mM EDTA, and 1% sodium azide). Cells were treated with Fc blocker (BD Biosciences) for 15 minutes and stained with FACS antibodies for 30 minutes at 4°C. After staining, cells were washed and resuspended in FACS buffer before applying to BD FACSCelesta (BD Biosciences). Result was analyzed by FlowJo v10 Software (BD Biosciences).

### Enzyme-linked immunosorbent assay

Detailed procedure is described in SI Appendix, Supplementary methods. Ninety-six-well ELISA plate (Greiner) coated with purified K8.1-Fc antigen was blocked with 3% BSA. Diluted mouse serum was added for 2 hours binding and washed with PBS-T. HRP-conjugated mouse IgG was then added for 90 minutes of incubation and washed with PBS-T. TMB substrate (3,3′,5,5′ – tetramethylbenzidine; BD Biosciences) was applied to each well and stopped by sulfuric acid. Absorbance was measured with optical density at 450 nm.

To determine the concentration of secreted IFN-γ and TNF-α, harvested media were applied to human IFN-γ or TNF-α quantikine ELISA Kit (R&D Systems) according to the manufacturer’s suggested protocol.

### Neutralization assay

Detailed procedure was described in SI Appendix, Supplementary methods. HEK293T or MC116 cells were seeded in 96-well-plate 24 hours or 1 hour before assay, respectively. Diluted serum from mouse was mixed with KSHV.219 and incubated at 37°C for 1 hour. Fifty microliter of the mixture was added into cells in 50 µL of culture media. Fifty microliter fresh culture medium was added the next day and incubated for another day. Cells were then washed and fixed with 1% paraformaldehyde (Sigma) in FACS buffer. Infectivity was determined by flow cytometry.

### T cell response

Detailed procedure was described in SI Appendix, Supplementary methods. Splenocyte was seeded in 96-well plate and treated with K8.1 overlapping peptide pool in the presence of BD GolgiPlug (BD Biosciences). Cells were harvested after 12 hours stimulation and followed with surface staining and intracellular staining to characterize induction of cellular immunity via flow cytometry.

### DNA extraction

#### Cell/splenocyte

Cells were resuspended in lysis buffer [NP-40 diluted to 1% (vol/vol) in PBS] and incubated at 4°C for 15 minutes. Insoluble debris was removed by centrifugation at 21,000 g for 15 minutes. DNA was extracted by phenol:chloroform:isoamyl alcohol (pH 8), 25:24:1 in (vol/vol) (Sigma). Nucleic acid in aqueous phase was precipitated with 100% ethanol and washed by 75% ethanol to remove residual salt. DNA pellet was dissolved in nuclease-free water.

#### Lung

DNA in lung tissues was extracted by DNA extraction kit (IBI Scientific) according to manufacture’s guideline. In briefly, lung tissue less than 25 mg in tube was added with 0.4 mL lysis buffer containing beta-mercaptoethanol (Sigma), and tissue was disrupted by Tissue Lyser (Qiagene) with frequency 30/second for 3 minutes. Homogenized tissue was centrifuge with 21,000 g for 15 minutes to remove debris, and DNA in supernatant was captured and isolated by columns provided by DNA extraction kits.

### RNA extraction and reverse transcription

Cells were harvested in 1 mL TRIzol (Sigma), and RNA was extracted by chloroform followed by phenol:chloroform:isoamyl alcohol (pH 4.5), 125:24:1 in (vol/vol) (Invitrogen). Nucleic acid in aqueous phase was precipitated with isopropanol and washed by 75% ethanol to remove residual salt. RNA pellet was dissolved in nuclease-free water. One microgram RNA was subjected to reverse transcription by iScript cDNA Synthesis Kit (Bio-Rad) in final 20 µL reaction.

### Real-time quantitative PCR

DNA was mixed with 0.6 mmol of primer and SsoAdvanced universal SYBR Green Supermix (Bio-Rad) in 15 µL. PCR reaction was performed by CFX96 Touch Real-Time PCR Detection System (Bio-Rad) with suggested protocol.

### IHC staining

Detailed procedure was described in SI Appendix, Supplementary methods. In short, slides were incubated at 72°C overnight and subjected to the process of dewaxing, rehydration, and antigen presentation. Endogenous peroxidase activity was quenched with 3% H_2_O_2_ (sigma) before blocking. Tissues were then stained with primary antibody, MHV68 ORF61, followed by secondary antibody. Images of stained slides were captured with slide scanner Aperio AT2 (Leica) and analyzed by QuPath (53).

### Statistical analysis

All asterisks in the figures indicate statistical significance between control and the respective immunized group. Statistical analysis was performed by GraphPad Prism with student’s t test (* indicates *P* < 0.05, ** indicates *P* < 0.01, *** indicates *P* < 0.005, and **** indicates *P* < 0.001).

## SUPPLEMENTAL MATERIAL

Fig. S1 mbio.02913-24-s0001.tif MHV68-K-K8.1 gene expression in immunized mice lung.

Legend mbio.02913-24-s0002.docx Figure S1 legend.

Supplemental text mbio.02913-24-s0003.docx Supplemental information for methods.

Table S1 mbio.02913-24-s0004.docx K8.1 peptide pool sequence.

Table S2 mbio.02913-24-s0005.docx Sequences for qPCR.

Table S3 mbio.02913-24-s0006.docx Antibody list for flow cytometry.

ASM does not own the copyrights to Supplemental Material that may be linked to, or accessed through, an article. The authors have granted ASM a non-exclusive, world-wide license to publish the Supplemental Material files. Please contact the corresponding author directly for reuse.
