# Supplementary figures and images for: Development of KSHV vaccine platforms and chimeric MHV68-K-K8.1 glycoprotein for evaluating the in vivo immunogenicity and efficacy of KSHV vaccine candidates
Source: mBio. 2024 Oct 30;15(12):e02913-24. doi: 10.1128/mbio.02913-24 (PMC11633179; doi:10.1128/mbio.02913-24)

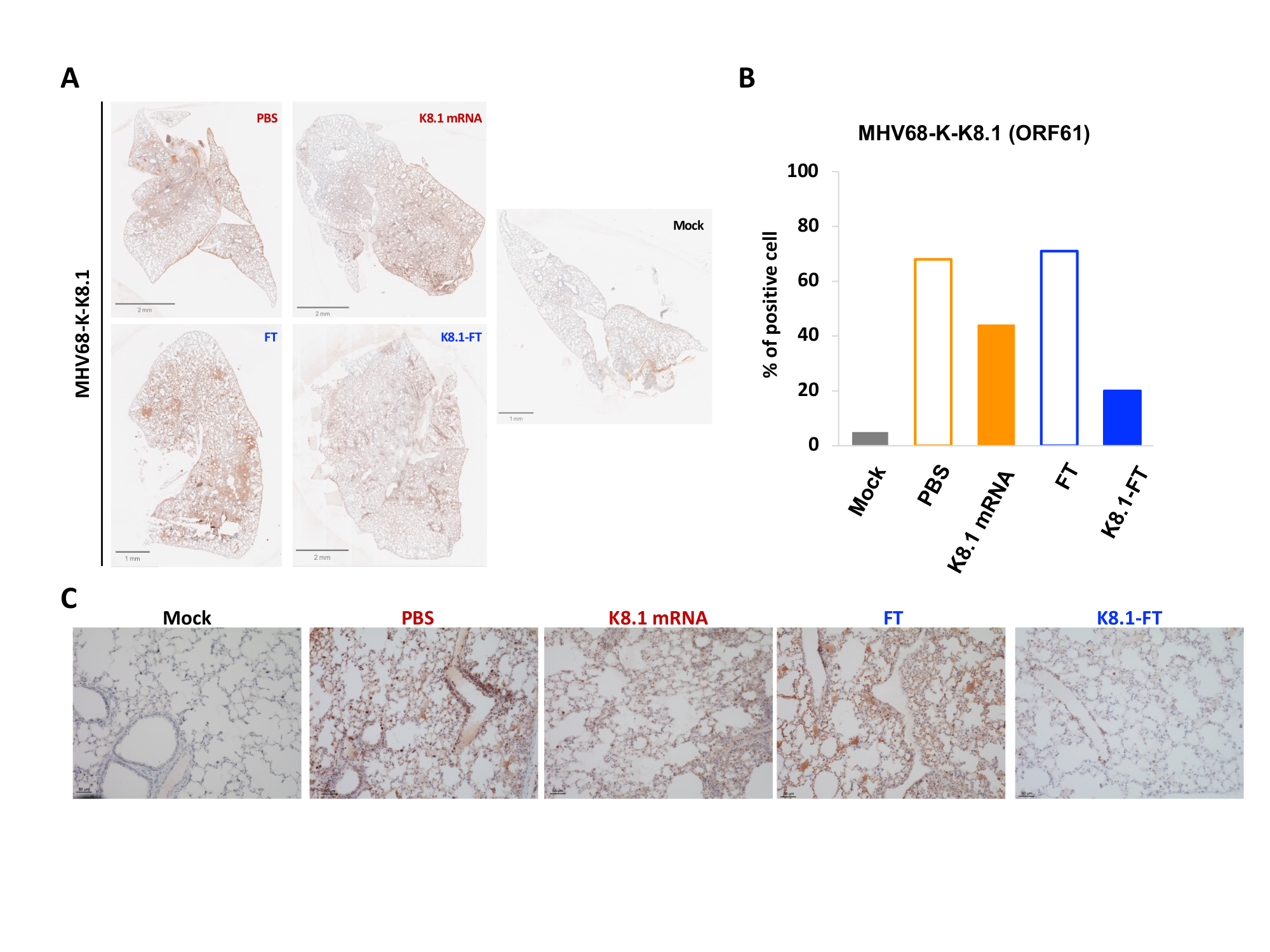

Supplement: Fig. S1 — MHV68-K-K8.1 gene expression in immunized mice lung. [file mbio.02913-24-s0001.tif]
