# Supplementary material for: Development of KSHV vaccine platforms and chimeric MHV68-K-K8.1 glycoprotein for evaluating the in vivo immunogenicity and efficacy of KSHV vaccine candidates
Source: mBio. 2024 Oct 30;15(12):e02913-24. doi: 10.1128/mbio.02913-24 (PMC11633179; doi:10.1128/mbio.02913-24)
Supplement: Legend — Figure S1 legend. [file mbio.02913-24-s0002.docx]

**Supplementary Figure**

**SI Appendix, Fig. S1 MHV68-K-K8.1 gene expression in immunized mice lung**

(A) Visualization of antigens from mice immunized with the vaccine candidates and infected with MHV68-K-K8.1 via MHV68 ORF61 antibody for tissue staining. Whole tissue section staining were acquired by slide scanner. (B) Quantify ORF61 positive-cell percentage in (A) by QuPath. (C) Representative enlarged images as described in (A) were acquired by microscope BX-X810 (Keyence)
