# Supplementary material for: Development of KSHV vaccine platforms and chimeric MHV68-K-K8.1 glycoprotein for evaluating the in vivo immunogenicity and efficacy of KSHV vaccine candidates
Source: mBio. 2024 Oct 30;15(12):e02913-24. doi: 10.1128/mbio.02913-24 (PMC11633179; doi:10.1128/mbio.02913-24)
Supplement: Supplemental text — Supplemental information for methods. [file mbio.02913-24-s0003.docx]

**Supplementary Method**

**BACmid engineering**

To replace MHV68 M7 with KSHV K8.1, K8.1 sequence was cloned into a kanamycine-resistant gene-containing vector, pEP-KanS. Fragments including the kanamycine resistant gene and K8.1 were amplified by PCR with primers covering homologous regions for specific locus recombination. To utilize two-step Red-mediated recombination for MHV68 genome engineering, MHV68 genomic bacterial artificial chromosome (BAC, MHV68 BAC WT) was extracted from *Escherichia coli* DH10B-MHV68 BAC and electroporated into *E.coli* GS1783 strain to express heat-inducible Red recombinase and arabinose-inducible I-SceI endonuclease. In the first recombination, PCR fragments were electroporated into heat-induced GS1783 (at 42^o^C) with MHV68 BAC WT and plated on LB agar supplemented with 30 ug/ml kanamycine (Sigma) and 30 ug/ml chloramphenicol (Sigma). To remove the kanamycin selective marker, another recombination was performed by using 1% arabinose (Sigma) at 30^o^C for 1 hour, followed by induction at 42^o^C 15 minutes. The resultant engineered construct was analyzed by sequencing. BACmid was also extracted, digested, and separated by agarose gel electrophoresis to confirm the expected fragmented pattern.

**Virion purification with sucrose gradient ultracentrifugation**

Vero E6 cells were infected with MHV68 WT or MHV68-K-K8.1 for two to four days to reach 90% cytopathic effect (CPE). Culture medium was harvested and centrifuged at 10,000 g for 20 minutes to remove cell debris. The supernatant was further centrifuge at 24,000 rpm for 1 hour at 4^o^C using SW32Ti rotor (Beckman Coulter). The pellet was resuspended in 150 ul DPBS (Gibco) and kept at 4^o^C for 12-16 hrs. To create a sucrose gradient, 5 ml of 15% sucrose and 5 ml of 50% sucrose were mixed in a 13.2 ml open-top ultra-clear tube (Beckman) using a Biocomp Gradient Master machine (Biocomp). The resuspended pellet was gently loaded on top of the sucrose gradient solution and centrifuged with SW41 rotor (Beckman) at 24,000 rpm for 1 hour at 4^o^C. The non-clear layer was collected in 10 ml DPBS and centrifuged again at 24,000 rpm for 1 hour at 4^o^C. The pellet was resuspended in DPBS and stored in -80^o^C.

**Plaque assay**

Vero E6 cells were seeded one day before infection with the virus. MHV68 viruses were diluted 10-fold from 10^0^ to 10^-6^ in DMEM. Vero E6 cells were then infected with the diluted virus and incubated at 37^o^C for 2 hours. Cells were washed with DPBS and overlaid with DMEM with 0.4% agarose (Lonza). The agarose mixture was left to solidify for 15 minutes at room temperature, and the cells were then returned to 37^o^C incubator with 5% CO_2_ until plaque formed. To fix and stain cells, 0.2% crystal violet (Sigma) in 20% ethanol was added to each well and incubate for 1 hour. The agarose overlay and dye were removed by tap water.

**K8.1 mRNA vaccine generation**

The K8.1 sequence in human codon was synthesized (GeneScript) and cloned into pZMV plasmid to generate pZMV-K8.1 Hm. Linearized pZMV-K8.1 by *Hpa*I was utilized as a template for *in vitro* transcription to generate K8.1 mRNA according to the manufacturer’s instruction (New England Biolab) in combination with 5 mM 1-Methyl-PseudoUridine (TriLink) and 4 mM CleanCap Reagent AG (TriLink) for co-transcriptional 5’ capping. After a 4-hour incubation at 37^o^C, the DNA template was digested by DNase I (Zymo Research) for 15 minutes at room temperature and RNA was purified using Monarch RNA cleanup kit (New England Biolab). Polyadenylation was completed using *E. coli* Poly (A) polymerase kit (New England Biolab) and the resultant RNA was purified with the RNA cleanup kit (New England Biolabs). RNA fragment size and integrity were checked by electrophoresis on a 1% denaturing agarose gel (with 1% formaldehyde) in MOPS buffer (Sigma).

RNA was encapsulated into lipid nanoparticle according to manufacture’s suggestions. In brief, 150 ug of K8.1 mRNA in PNI buffer was mixed with GenVoy-ILM diluted in ethanol at a 3:1 ratio by NanoAssemblr Ignite microfluidic instrument (Precision Nanosystems). After encapsulation, RNA-LNP was diluted 40-fold in PBS (Gibco) and concentrated using a 10 kDa cutoff Amicon Ultra Centrifugal filter (Sigma). To sterilize mRNA-LNP, it was filtered through 0.2 um pore-sized filter (Pall). RNA concentration and encapsulation efficiency were determined using the Ribogreen RNA assay kit (Invitrogen).

**K8.1_26-87_-ferritin (FT) vaccine purification**

HEK293T cells were transfected with Ferritin or K8.1_26-78_-FT expression construct. One day after transfection, the culture medium was replaced by FreeStyle 293 Expression Medium (Gibco) and harvested three days after changing the medium. Cell debris was removed using 0.22 um filter before the medium was further purified via Akta pure chromatography system (Cytiva). K8.1_26-78_-FT was purified from the medium using HiTrap Q Fast Flow (Cytiva) with Tris-HCl buffer (pH 7.5) in gradient increase from 0 M to 1 M NaCl. Fractions containing K8.1_26-87_-FT nanoparticles were identified using SDS-PAGE and Coomassie blue staining, concentrated, and buffer exchanged with a 100 kDa Amicon Ultra centrifugal filter (Millipore).

**K8.1_26-198_-Fc purification**

HEK293T cells were transfected with the Fc or K8.1_26-198_-Fc expression construct. One day after transfection, the culture medium was replaced with FreeStyle 293 Expression Medium (Gibco) and harvested three days after changing the medium. Cell debris in the medium was removed by centrifugation at 800 g for 15 minutes. Fc or K8.1_26-198_-Fc was precleared with Sepharose beads (Cytiva) and purified with protein A/G Agarose beads (Thermo Fisher). Protein on beads was eluted with 2.7 ml elution buffer (0.1 M Glycine-HCl, pH 2.7) and neutralized with 0.3 ml neutralization buffer (1 M Tris-HCl, pH 9). The eluents were dialyzed against PBS overnight at 4^o^C.

**Enzyme-Linked Immunosorbent Assay (ELISA)**

To determine anti-K8.1 antibody titer in mouse serum after immunization, 100 ng of the purified K8.1-Fc was coated in each well of a 96-well plate (Greiner) for 12 to 18 hours at 4^o^C. Each well was blocked with 3% bovine serum albumin (BSA; Sigma) in PBS for 2 hours at room temperature. Diluted mouse serum samples were added to each well for incubation for 2 hours on an orbital shaker and washed 3 times with PBS-T (0.05% Tween-20). HRP-conjugated mouse IgG antibody was then added to each well for a 90-minute incubation on an orbital shaker and washed 3 times with PBS-T. 100 ul of TMB substrate (3,3′,5,5′ – tetramethylbenzidine; BD Biosciences) was applied to each well and incubated at room temperature for seven to eight minutes. 50 ul of 2N sulfuric acid was added to each well to stop the color development and the absorbance values were determined by measuring optical density (OD) at 450 nm. The endpoint titer was determined as dilution point that absorbance exceeded three times the absorbance of the background wells.

**Neutralization assay**

1x10^4^ HEK293T cells were seeded in a 96-well-plate one day before assay. 5x10^4^ MC116 cells were plated in a 96-well-plate on the day of the assay. Serum samples from mice were diluted 450-fold with complete DMEM for HEK293T cells or complete RPMI for MC116 cells. The virus was added to the diluted serum at a 1:1 ratio and incubated at 37^o^C for one hour. After incubation, 50 ul of the mixture was added to the HEK293T or MC116 cells in 50 ul culture medium. The next day, 50 ul of culture medium was added to the cells and incubated for another day. Cells were washed and fixed with 1% paraformaldehyde (Sigma) in FACS buffer. The percentage of KSHV-infected cells was analyzed by flow cytometry. Infectivity was calculated as (GFP% _PBS or FT control_ – GFP% _K8.1_ _immunized serum_)/GFP% _PBS or FT control_ X 100%. 100% infection was defined as full infectivity of the virus incubated with mock serum.

**T cell response**

5x10^5^ splenocytes were seeded in a 96-well-plate in the presence of 1 ug / ml of K8.1-covering overlapping peptide pool, OLP (GeneScript) with or without 1 ul/ml BD GolgiPlug (BD Biosciences). After 12 hours of incubation at 37^o^C, cells were harvested, washed with FACS buffer, blocked with BD Fc block (BD Biosciences), and stained with antibodies, including anti-CD3-BV786, CD4-APC and CD8-BV421 antibodies (BioLegend). Cells were further permeabilized using the BD Cytofix/Cytoperm Plus Fixation/Permeabilization Kit (BD Biosciences) according to the manufacturer’s suggested protocol. Intracellular cytokines were stained with anti-IFN-γ-FITC and TNF-α-PE antibodies (BioLegend). After staining, cells were washed and resuspended in FACS buffer. Fluorescence signals were analyzed using BD FACSymphony and FlowJo software. For secreted cytokine level analysis, culture medium from splenocytes stimulated with K8.1-OLP in the absence of BD GolgiPlug were collected and analyzed by ELISA (R & D systems) and cytokine array with Target 48 Mouse Cytokine panel (Olink).

**IHC staining**

Paraffin embedded tissue sections were incubated overnight at 72^o^C before dewaxing with xylene. The tissue was sequentially rehydrated in 100%, 95%, 80% and 70% ethanol. After boiling the sections for 30 minutes in sodium citrate buffer (sigma), the samples were cooled down for one hour at room temperature, then treated with 3% H_2_O_2_ (Sigma) for five minutes and blocked with 5% normal goat serum (Vector). The primary antibody recognizing MHV68 ORF61 was diluted at a 1:500 ratio in PBS, added to the tissue sections, and incubated overnight at 4^o^C. The secondary antibody, biotinylated anti-rabbit IgG antibody (Vector), and signal development (Vector) were conducted following the manufacture’s instructions. Cell nuclei were stained with hematoxylin (Vector) and the tissue was soaked in acid alcohol (0.125% HCl in ethanol), 0.5% lithium carbonate (in water), and dehydrated with 70%, 80%, 95%, 100% Ethanol and xylene in series. Tissue was mounted with mounting media (Epredia) and covered with cover slide. Stained images were captured with slide scanner Aperio AT2 (Leica) and analyzed by QuPath software.
