# Supplementary material for: Development of KSHV vaccine platforms and chimeric MHV68-K-K8.1 glycoprotein for evaluating the in vivo immunogenicity and efficacy of KSHV vaccine candidates
Source: mBio. 2024 Oct 30;15(12):e02913-24. doi: 10.1128/mbio.02913-24 (PMC11633179; doi:10.1128/mbio.02913-24)
Supplement: Table S1 — K8.1 peptide pool sequence. [file mbio.02913-24-s0004.docx]

**Supplementary Table**

**Table 1 K8.1 peptide pool sequence**

| MSSTQIRTEIPVALL | IRTEIPVALLILCLC | ILCLCLVACHANCPT |
| --- | --- | --- |
| LVACHANCPTYRSHL | ANCPTYRSHLGFWQE | YRSHLGFWQEGWSGQ |
| GFWQEGWSGQVYQDW | GWSGQVYQDWLGRMN | VYQDWLGRMNCSYEN |
| LGRMNCSYENMTALE | CSYENMTALEAVSLN | MTALEAVSLNGTRLA |
| AVSLNGTRLAAGSPS | GTRLAAGSPSSEYPN | AGSPSSEYPNVSVSV |
| SEYPNVSVSVEDTSA | VSVSVEDTSASGSGE | EDTSASGSGEDAIDE |
| SGSGEDAIDESGSGE | DAIDESGSGEEERPV | SGSGEEERPVTSHVT |
| EERPVTSHVTFMTQS | TSHVTFMTQSVQATT | FMTQSVQATTELTDA |
| VQATTELTDALISAF | ELTDALISAFSGSYS | LISAFSGSYSSGEPS |
| SGSYSSGEPSRTTRI | SGEPSRTTRIRVSPV | RTTRIRVSPVAENGR |
| RVSPVAENGRNSGAS | AENGRNSGASNRVPF | NSGASNRVPFSATTT |
| NRVPFSATTTTTRGR | SATTTTTRGRDAHYN | TTRGRDAHYNAEIRT |
| DAHYNAEIRTHLYIL | AEIRTHLYILWAVGL | LLGLVLILYLCVPRC |
| LILYLCVPRCRRKKP | YLCVPRCRRKKPYIV |  |
