# Supplementary material for: Development of KSHV vaccine platforms and chimeric MHV68-K-K8.1 glycoprotein for evaluating the in vivo immunogenicity and efficacy of KSHV vaccine candidates
Source: mBio. 2024 Oct 30;15(12):e02913-24. doi: 10.1128/mbio.02913-24 (PMC11633179; doi:10.1128/mbio.02913-24)
Supplement: Table S2 — Sequences for qPCR. [file mbio.02913-24-s0005.docx]

**Supplementary Table**

**Table 2 sequence for qPCR**

| Target (DNA) | Forward Sequence | Reverse Sequence |
| --- | --- | --- |
| Orf50 | AGAAACCCACAGCTCGCACTT | CAATATGCTGGACAGGCGTATC |
| Orf73 | TAGATCCAGGTGATCCTGTGGC | CCGCATAATCCATCTGATCCAT |
| L8 | CATCCCTTTGGAGGTGGTA | CATCTCTTCGGATGGTGGA |
| Target (cDNA) | Forward Sequence | Reverse Sequence |
| ORF 4 | GGGATTGTGGGTGTAAATGGTGAC | CAGGGAGTCACGGTTGTCCA |
| ORF 6 | AGGGACAGATTTCCTCAGGTGC | CTGGCGTGGAAGCTGTTACC |
| ORF 8 (gB) | GGCCCAAATTCAATTTGCCT | CCCTGGACAACTCCTCAAGC |
| ORF 9 | CAATTGCTGTATCCCATCTGCG | GGAAACCCACATTCACCCAAAC |
| ORF 50 | GGCCGCAGACATTTAATGAC | GCCTCAACTTCTCTGGATATGCC |
| ORF 57 | GCTAGACGAACAGTTGGCCTG | TTGGTGCGGTGTGTTTGTCC |
| ORF 65 (M9) | AGACAGGGTCCATCATTTTGGC | TTGGCAAAGACCCAGAAGAAGC |
| ORF 73 | AAGGGTTGTCTTGGCCTACTGTG | AGAGATGCTGTGGGACCATGTTG |
| ORF75C | ATTCCAGAGTATTCGTTCAG | GTCAGCACTGTCCAATTCT |
| L8 | CATCCCTTTGGAGGTGGTA | CATCTCTTCGGATGGTGGA |
