# Supplementary material for: Development of KSHV vaccine platforms and chimeric MHV68-K-K8.1 glycoprotein for evaluating the in vivo immunogenicity and efficacy of KSHV vaccine candidates
Source: mBio. 2024 Oct 30;15(12):e02913-24. doi: 10.1128/mbio.02913-24 (PMC11633179; doi:10.1128/mbio.02913-24)
Supplement: Table S3 — Antibody list for flow cytometry. [file mbio.02913-24-s0006.docx]

**Supplementary Table**

**Table 3 Antibody list for flow cytometry**

| Antibody | Vendor | Catalog number |
| --- | --- | --- |
| FITC anti-mouse IFN-γ Antibody | BioLegend | 505806 |
| PE anti-mouse TNF-α Antibody | BioLegend | 506306 |
| Brilliant Violet 785™ anti-mouse CD3 Antibody | BioLegend | 100231 |
| APC anti-mouse CD4 Antibody | BioLegend | 100516 |
| CD8a Monoclonal Antibody (53-6.7), eFluor™ 450 | eBioscience | 48-0081-82 |
